# Integrated Metabolomic and Transcriptomic Analysis Reveal the Production Mechanism of Semicarbazide in *Macrobrachium rosenbergii* Under Urea Conditions

**DOI:** 10.3390/foods13233817

**Published:** 2024-11-27

**Authors:** Jun Li, Di Wang, Shengjun Chen, Fangfang Gao, Chunsheng Li, Yang Feng, Jianchao Deng

**Affiliations:** 1 Guangdong Provincial Key Laboratory of Lingnan Specialty Food Science and Technology, Key Laboratory of Green Processing and Intelligent Manufacturing of Lingnan Specialty Food, Ministry of Agriculture and Rural, College of Light Industry and Food, Zhongkai University of Agriculture and Engineering, Guangzhou 510225, China; lijun@zhku.edu.cn; 2 Key Laboratory of Aquatic Product Processing, Ministry of Agriculture and Rural Affairs, National R&D Center for Aquatic Product Processing, South China Sea Fisheries Research Institute, Chinese Academy of Fishery Sciences, Guangzhou 510300, China; wangdi@scsfri.ac.cn (D.W.); gff2201718230@163.com (F.G.); lichunsheng@scsfri.ac.cn (C.L.); fengyang@scsfri.ac.cn (Y.F.); dengjianchao@scsfri.ac.cn (J.D.); 3 Key Laboratory of Efficient Utilization and Processing of Marine Fishery Resources of Hainan Province, Sanya Tropical Fisheries Research Institute, Sanya 572018, China

**Keywords:** *Macrobrachium rosenbergii*, endogenous semicarbazide, urea, metabolomic, transcriptomic

## Abstract

Semicarbazide (SEM) is commonly utilized as a biomarker for detecting the usage of nitrofurazone (NFZ); however, its endogenous presence in aquatic products complicates detection and poses challenges to the quality and safety of these products. Although previous research suggests a potential link between SEM and urea, the specific mechanisms underlying its production under induced conditions remain unclear. To solve the above problem, the integrated metabolomic and transcriptomic analyses were performed for systematically exploring endogenous production mechanisms underlying SEM in *Macrobrachium rosenbergii* under urea conditions. As a result, urea exposure significantly disrupted key pathways, including glycine, serine, and threonine metabolism; 2-oxocarboxylic acid metabolism; and protein digestion and absorption, thereby highlighting the role of amino acid metabolism in SEM formation. Compared to traditional single-omics approaches, this method provided a comprehensive analysis of gene–metabolite interactions, revealing the mechanism of endogenous production of SEM in *M. rosenbergii.* This research offers new insights into enhancing aquatic product safety and quality and represents a methodological reference for future research regarding the endogenous SEM production mechanisms.

## 1. Introduction

Semicarbazide (SEM) serves as the biomarker for detecting nitrofurazone (NFZ), an antibacterial agent that was once widely applied in agriculture. NFZ proved highly effective in preventing bacterial infections in livestock and aquatic products, contributing to enhanced productivity and reduced losses due to disease [1]. However, since the 1990s, NFZ has been banned in several countries due to its carcinogenic properties and other adverse effects, such as teratogenicity and mutagenicity, which pose significant risks to human health [2]. Despite the ban, SEM is detected from numerous food products that do not use NFZ, including honey, bread flour, shrimp, and seaweed [3,4,5].

Crustacean aquatic products are highly valued by consumers for their high protein content, low fat content, and rich vitamin and mineral profile, making them an important source of nutrition worldwide. However, over the past two decades, 19.5% of crustacean aquatic products have been found to contain SEM, despite the absence of NFZ usage during aquaculture [6]. Saari et al. were the first to suggest that endogenous SEM may exist in crustacean products, and this endogenous SEM affects the safety and marketability of such products, particularly in regions with stringent regulatory standards for chemical residues [7]. Endogenous SEM makes it even complicated to monitor the safety and quality in aquatic products, as it creates challenges in distinguishing between illicit NFZ addition and natural SEM formation, thereby posing potential risks to product quality and the aquaculture industry [8,9]. Therefore, elucidating the mechanisms of endogenous SEM formation is crucial for enhancing the quality assessment and safety of aquatic products, as well as for developing more effective regulatory policies. Research indicates that the mechanisms underlying endogenous SEM formation involve complex metabolic pathways. Hoenicke et al. proposed that endogenous SEM may be produced through enzymatic activities in the urea cycle, involving compounds like arginine, citrulline, histidine, urea, and creatinine, which are common metabolic intermediates in crustaceans [10]. Additionally, hydrazine in the urea cycle may react with nitrogenous substances to form SEM, further complicating the understanding of its biochemical origins [11]. Yu et al. suggested that SEM formation could be related to urea present in food [4]. The widespread application of urea as the nitrogen fertilizer in agriculture results in higher urea contents within estuarine and coastal waters [12], and it is also utilized as a fertilizer in aquaculture to improve water quality and promote plankton growth [13]. This has led to a significant rise in environmental nitrogen levels [14], which could indirectly contribute to SEM formation in aquatic organisms. However, despite studies suggesting that urea exerts an important effect on SEM formation, the specific metabolic pathways and their impact on *Macrobrachium rosenbergii* remain unclear [15]. Therefore, more studies are warranted for elucidating the molecular mechanisms of endogenous SEM formation.

Omics technologies have emerged as powerful tools in recent years. For instance, metabolomics is a highly valuable approach for investigating molecular mechanisms related to phenotypic traits. Metabolomic analysis has revealed that the primary reason for the survival of *Exopalaemon carinicauda* in high-carbonate environments is predominantly linked to lipid metabolism [16]. Transcriptomics, on the other hand, refers to the comprehensive analysis of mRNA expression within a biological system, facilitating a deeper understanding of the mechanisms underlying toxicity and stress [17]. The integration of transcriptomics and metabolomics has become a popular strategy. Qiao et al. employed both metabolomics and transcriptomics to examine the impact of copper exposure on pig kidneys, identifying molecular targets associated with energy metabolism and oxidative stress, thereby providing new insights into the toxicological effects of heavy metals [17]. This multi-omics approach is particularly useful for investigating and understanding the molecular mechanisms involved in specific environmental conditions, which aligns with our objective of identifying the molecular pathways implicated in SEM formation.

In this study, we first investigated the changes in SEM content in *M. rosenbergii* in presence of urea. We subsequently conducted untargeted metabolomic and transcriptomic analysis on the elucidating mechanisms of endogenous SEM production within *M. rosenbergii* under urea conditions. Our results shed new light on molecular mechanisms underlying the urea-induced production of endogenous SEM in *M. rosenbergii*.

## 2. Materials and Methods

### 2.1. Experimental Animals

*M. rosenbergii* (body weight: 45.0 ± 2.0 g) were provided by the aquaculture base of Shenzhen Research Center, South China Sea Fisheries Research Institute. Before this experiment, the shrimps were acclimated to the ambient conditions for 24 h; while acclimating, the fecal matter was removed by siphoning, and any visually defective shrimp were discarded. The 180 shrimp without visible defects were subsequently randomly and equally distributed into three groups: a control group, a group exposed to 50 mg/kg urea, and a group exposed to 80 mg/kg urea. The shrimp were then randomly assigned to nine fiberglass tanks, with each tank housing 20 shrimp, resulting in a culture density of about 30 tails/m^3^. The tanks were prefilled with fresh water or water containing urea, with a water temperature range of 25–29 °C, a pH of 7.8, and a dissolved oxygen (DO) level at 7.2 mg/L. To reduce the effect of urea on DO levels, fresh air was continuously supplied to the water via an electrogenic air pump throughout the experiment, ensuring that the DO value remained above 6.0 mg/L. Every animal experiment was conducted following relevant guidelines and gained approval from the ethics committee of Key Laboratory of Aquatic Product Processing, Ministry of Agriculture and Rural Affairs of China, South China Sea Fisheries Research Institute (approval date 15 July 2023).

### 2.2. SEM Detection

SEM was detected after optimizing the national standards [18]. The samples of *M. rosenbergii* were subjected to homogenization within the blender. Then, the homogenized mixture (2 g) was subjected to 100 ng mL^−1 13^C-^15^N_2_-SEM (0.1 mL) and 0.2 mol L^−1^ HCl (10 mL) using 0.05 mol L^−1^ 2-NBA (0.2 mL within methanol). After thorough mixing, the resulting mixture was incubated in a constant temperature oscillator under 37 °C and 200 rpm in the dark for 16 h. Subsequently, we added dipotassium hydrogen phosphate (2–3 mL) into this mixture for adjusting pH to 7.26–7.45. We also used 6 mL ethyl acetate to extract the derivatives, with two repetitions (15 min per repetition), and later centrifuged for a 10 min duration at 1872× *g*. Thereafter, we pooled ethyl acetate fractions and evaporated the solvent under 40 °C using nitrogen. The residue obtained was redissolved into the solution containing 0.1% formic acid–acetonitrile (1 mL), with the fat present being eliminated by introducing n-hexane (1.5 mL). The samples underwent 15 min of centrifugation at 10,278× *g* to collect supernatants, which were then filtered with the 0.22 μm filter in UPLC-MS/MS.

A C_18_ column (50 mm × 2.1 mm × 1.7 μm; Thermo, Waltham, MA, USA) was utilized to separate the final extracts. Typically, the mobile phase included 0.1% (*v*/*v*) formic acid, 5 mmol/L ammonium formate (A), and acetonitrile (B), which was eluted at the 0.35 mL/min constant flow rate with a gradient. The gradient elution procedure is shown in Table S1. We operated this mass spectrometer in the electrospray positive ion (ESI+) mode under the following parameters, multiple-reaction monitoring (MRM) mode, ion source temperature at 120 °C, desorption temperature at 600 °C, dissolvent gas flow rate (nitrogen) at 1000 L/h, and cone–hole gas flow rate (argon) at 50 L/h. SEM concentration was used to be horizontal coordinate, whereas the peak area ratio of SEM to ^13^C-^15^N_2_-SEM was adopted as vertical coordinate to establish the standard curve for SEM concentration. The parent ion, daughter ion, cone-hole voltage, and collision energy can be observed from Table S2.

### 2.3. Metabolomics Analysis

The control and 80 mg/kg urea group sample (100 g) that were rapidly immersed into liquid nitrogen for freezing were ground to fine powder with a mortar and pestle. Subsequently, the cold methanol/acetonitrile/water solution (1 mL, 2:2:1, *v*/*v*) was introduced and vortex-mixed. The mixture underwent 30 min of sonication on ice, followed by 20 min of centrifugation at 14,000× *g* and 4 °C to harvest supernatants in vacuum-drying. For mass spectrometry (MS), a mixture containing acetonitrile and water (100 μL, 1:1, *v*/*v*) was introduced for reconstitution, prior to vortexing as well as centrifugation at 14,000× *g* and 4 °C for 5 min to harvest supernatants for UHPLC analysis (1290 Infinity LC, Agilent Technologies, Santa Clara, CA, USA) using the ACQUIY UPLC BEH Amide column (2.1 mm × 100 mm, 1.7 µm; waters, Wexford, Ireland) under 25 °C. The mobile phase comprised A = 25 mM ammonium acetate and 25 mM ammonium hydroxide within water as well as B = acetonitrile. The following elution conditions were set as follows: 0–0.5 min, 95% B; 0.5–7 min, 95–65% B; 7–8 min, 65–40% B; 8–9 min, 40% B; 9–9.1 min, 40–95% B; and 9.1–12 min, 95% B. Injection volume and flow rate were 2 μL and 0.5 mL/min, respectively.

MS analysis was completed with quadrupole time-of-flight (AB Sciex TripleTOF 6600) through electrospray ionization (ESI) under positive/negative ion modes. ESI source parameter settings included: nebulizer gas (Gas1), 60 psi; auxiliary gas (Gas2), 60 psi; curtain gas (CUR), 30 psi; spray voltage (ISVF) ±5500 V (positive/negative modes); ion source temperature, 600 °C; first-level mass-to-charge ratio (m/z) detection range, 60–1000 Da; the second-level fragment ion m/z detection range, 25–1000 Da; and first- and second-level mass spectrum scan accumulation times of 0.20 and 0.05 s/spectrum, respectively. Second-level MS was conducted through information-dependent acquisition (IDA) in the high sensitivity mode chosen. Relevant parameters included collision energy at 35 ± 15 eV and declustering potential at ±60 V and excluded isotopes at 4 Da; ten candidate ions were monitored per cycle.

We transformed raw MS data into MzXML files with ProteoWizard. XCMS was conducted for aligning peaks, correcting retention time, and extracting peak areas. Subsequently, data underwent identification, preprocessing, quality evaluation, and data analysis [19].

### 2.4. Transcriptome Analysis

By utilizing TRIzol (Invitrogen, Waltham, MA, USA), total RNA extraction was completed in line with specific protocol. Thereafter, RNA quality and content were determined with Nanodrop 2000 spectrophotometer and the Qubit 2.0 fluorometer using Qubit RNA Assay Kit (Life Technologies, Carlsbad, CA, USA). The establishment and sequencing of cDNA libraries were completed using the Illumina HiSeq 2500 platform. After raw reads were obtained, the data were processed for removing adapter sequences, low-quality reads and those including poly-N sequences were discarded. After assembly with Trinity [20], the clean reads were annotated into databases, like the Nr, Pfam, SwissProt, GO, and KEGG databases. Unigene expression was analyzed through fragments per kilobase per million reads (FPKM) [21]. DESeq2 software (http://bioconductor.org/packages/stats/bioc/DESeq2/, accessed on 24 November 2024) was applied in analyzing differentially expressed genes (DEGs) between urea and control conditions, with significant DEGs distinguished by FDR ≤ 0.05 and |log2 (fold-change)| ≥ 1. The functional annotations of DEGs were examined with GO and KEGG databases.

### 2.5. Gene Expression Verification Through RT-Qpcr

We randomly selected eight DEGs to conduct real-time quantitative PCR (RT-qPCR) analysis for further analyzing transcriptomic data. Table S6 shows primers utilized in the present work. SYBR Green method (SYBR^®^ Green Real-time PCR Master Mix Plus, TOYOBO, Osaka, Japan) was employed in RT-qPCR using an ABI 7500 system (ABI, Los Angeles, CA, USA). The reaction volume consisted of SYBR Green Real-time PCR Master Mix Plus (10 μL), diluted cDNA (2 μL), respective primers (0.8 μL), and DNase-free water (6.4 μL). The description of the RT-qPCR process can be found in another study [22]. We adopted the 2^−ΔΔCt^ method to quantify gene expression.

### 2.6. Statistical Analysis

Every experiment was carried out thrice. GraphPad Prism 10.0 (GraphPad Software, San Diego, CA, USA) was adopted for data analysis. Tukey’s HSD was used in statistical analyses, and data were represented by the mean ± standard deviation. *p* < 0.05 suggested significant differences.

## 3. Results

### 3.1. SEM Concentration of M. rosenbergii Under Urea Conditions

**Figure 1:**
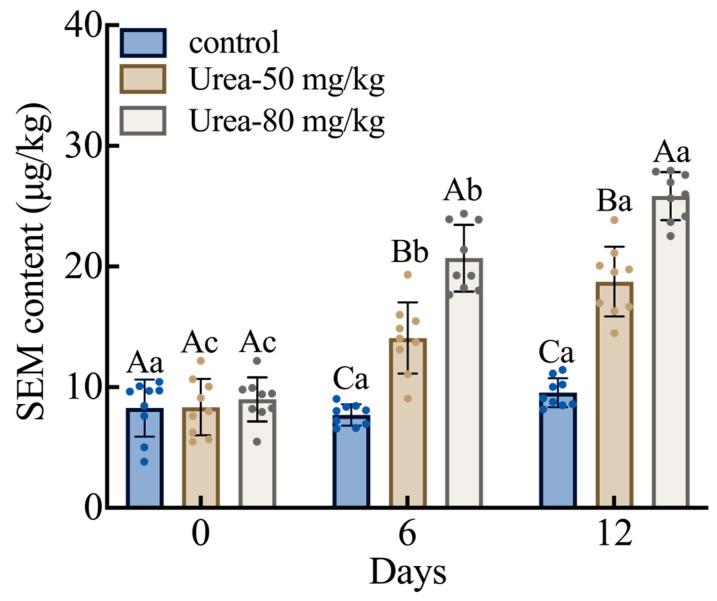
Content of SEM in *M. rosenbergii* under urea condition. Diverse uppercase letters stand for significant differences on each day (*p* < 0.05); lowercase letters are indicative of significant differences on diverse stress days at an identical stress concentration (*p* < 0.05).

Physiological experiments were conducted to evaluate the changes in the SEM concentration of *M. rosenbergii* under urea conditions (Figure 1). The SEM concentration in *M. rosenbergii* increased under urea conditions throughout the stress period. A dose-dependent relationship was observed, with the SEM concentration increasing with an increase in urea concentration. SEM concentration on day 0 was not significantly different between different groups. In contrast, on days 6 and 12, the SEM concentration increased remarkably in the urea-treated versus the control groups (*p* < 0.05). SEM concentration increased by 45.3% and 49.1% in the 50 mg/kg urea group and by 62.7% and 63.0% in the 80 mg/kg urea group on days 6 and 12, respectively.

### 3.2. Effects of Urea on the Metabolites in M. rosenbergii

**Figure 2:**
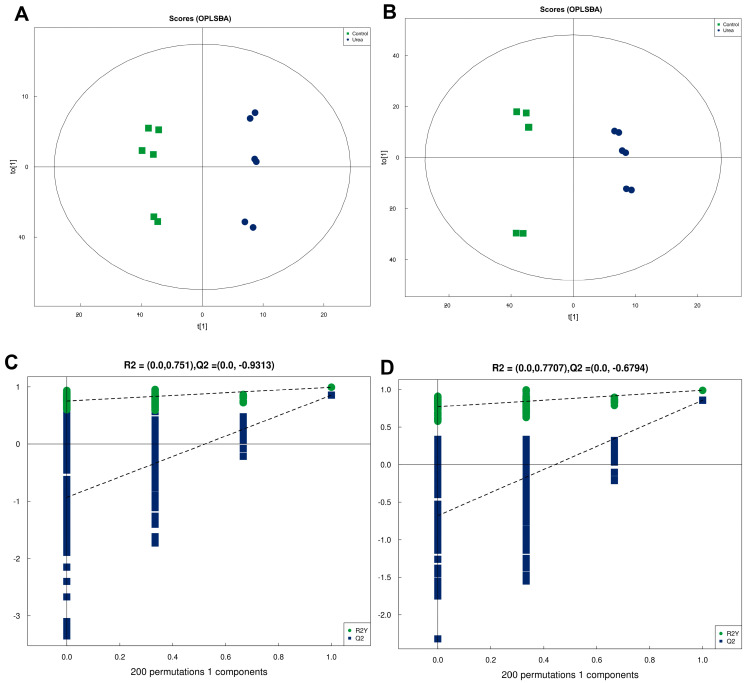
Orthogonal partial least-squares discriminant analysis (OPLS-DA) score plots (**A**,**B**) and OPLS-DA permutation plots (**C**,**D**) for *M. rosenbergii* under urea conditions in positive (**A**,**C**) and negative (**B**,**D**) ion modes.

To elucidate the mechanism of SEM production in *M. rosenbergii* under urea conditions, we performed metabolic profiling of the urea (80 mg/kg) and control groups using non-targeted metabolomics methods. The OPLS-DA of metabolites revealed that the urea group was well separated from control group (Figure 2A,B) [23]. The R2Y value was >0.9, while the Q2 values were above 0.5, indicating a high level of predictability within the model. Based on permutation test analysis, as the retention of replacement decreased, R2 and Q2 in stochastic model declined, which indicated the absence of model overfitting (Figure 2C,D).

**Figure 3:**
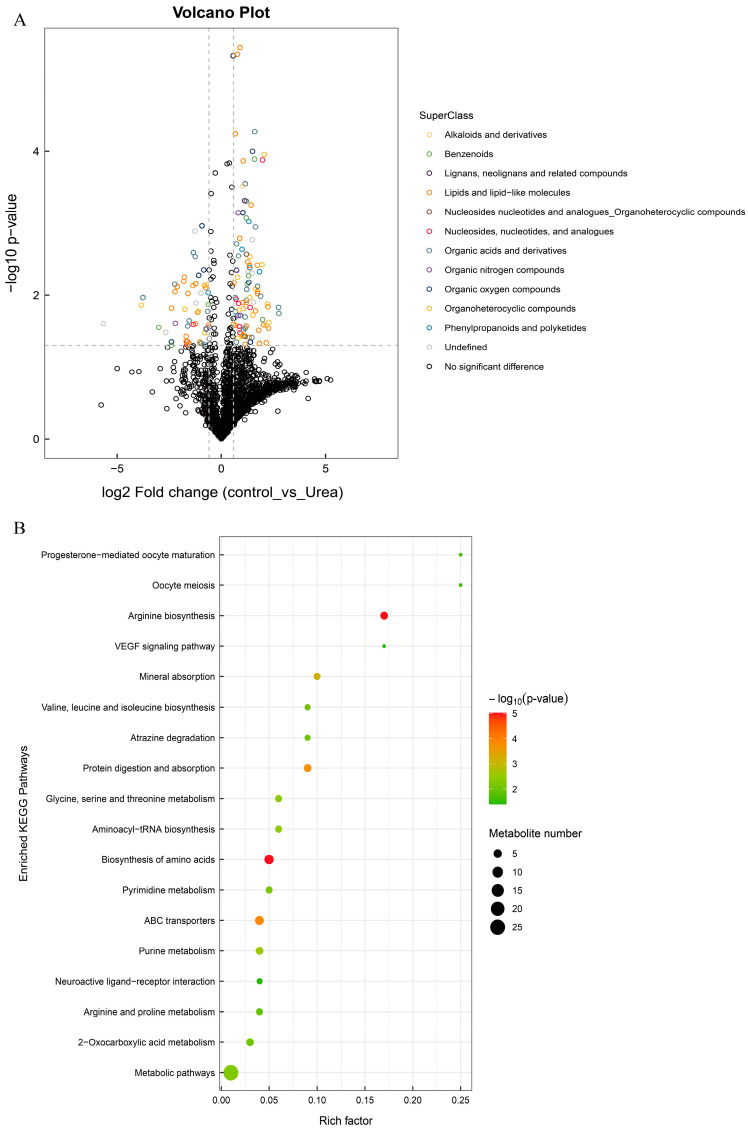
Volcano plot of DMs in *M. rosenbergii* under urea conditions (**A**); KEGG pathway analysis of DMs in *M. rosenbergii* under urea conditions (**B**).

Based on the threshold VIP > 1 and *p* < 0.05, we obtained 77 significantly differential metabolites (DMs) in urea versus control groups, including 33 upregulated metabolites and 44 downregulated metabolites. The 77 DMs were classified in the HMDB, and numerous abundant DMs were categorized as lipids, lipid-like molecules, and organic acids and their derivatives in the HMDB superclass (Figure 3A and Table S3). The KEGG enrichment was performed in order to investigate the urea-affected metabolic pathways. The 20 most enriched pathways can be observed in Figure 3B. In addition, the significance of 18 metabolic pathways was determined under urea conditions, and *p* < 0.5 indicates significant differences. KEGG pathway enrichment was conducted to map DMs onto metabolic pathways, and they were enriched primarily in the biosynthesis of amino acids (seven differentially abundant metabolites), ABC transporters (six differentially abundant metabolites), purine metabolism (four differentially abundant metabolites), protein digestion and absorption (four differentially abundant metabolites), arginine biosynthesis (four differentially abundant metabolites), and 2-oxocarboxylic acid metabolism, which were associated with most of the differentially abundant metabolites.

### 3.3. Effects of Urea Conditions on Gene Expression in M. rosenbergii

**Figure 4:**
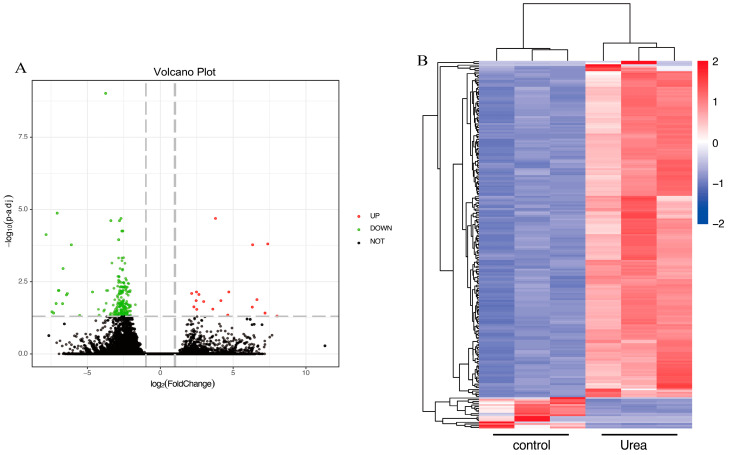
Volcano plot of DEGs in *M. rosenbergii* under urea conditions (**A**); hierarchical clustering of DEGs in the Control versus the urea condition groups (**B**).

To elucidate the mechanism underlying SEM production under urea conditions in *M. rosenbergii*, a comparative transcriptome analysis was conducted using RNA sequencing. In all groups, the Q20 and Q30 values were >98% and 95%, respectively, and genes mapped were greater than 70%, suggesting that our sequencing data were creditable (Table S4) [23]. In total, 212 DEGs were obtained in control versus urea-treated groups, of which 194 were downregulated and 18 were upregulated (Table S5). This finding was similar to that of another study in which there were more significantly downregulated genes than upregulated genes under environmental stress in *M. rosenbergii* because the biological functions of these genes were disrupted [22]. We used volcano plots (Figure 4A) to illustrate DEGs distribution between the urea and control groups. To visualize differential expressions in the control versus the urea groups, hierarchical cluster analysis was performed, and the results are displayed in a heat map (Figure 4B). Gene expression was significantly different in control versus urea groups (Figure 4A,B), which indicated that urea significantly affects gene expression in *M. rosenbergii*.

**Figure 5:**
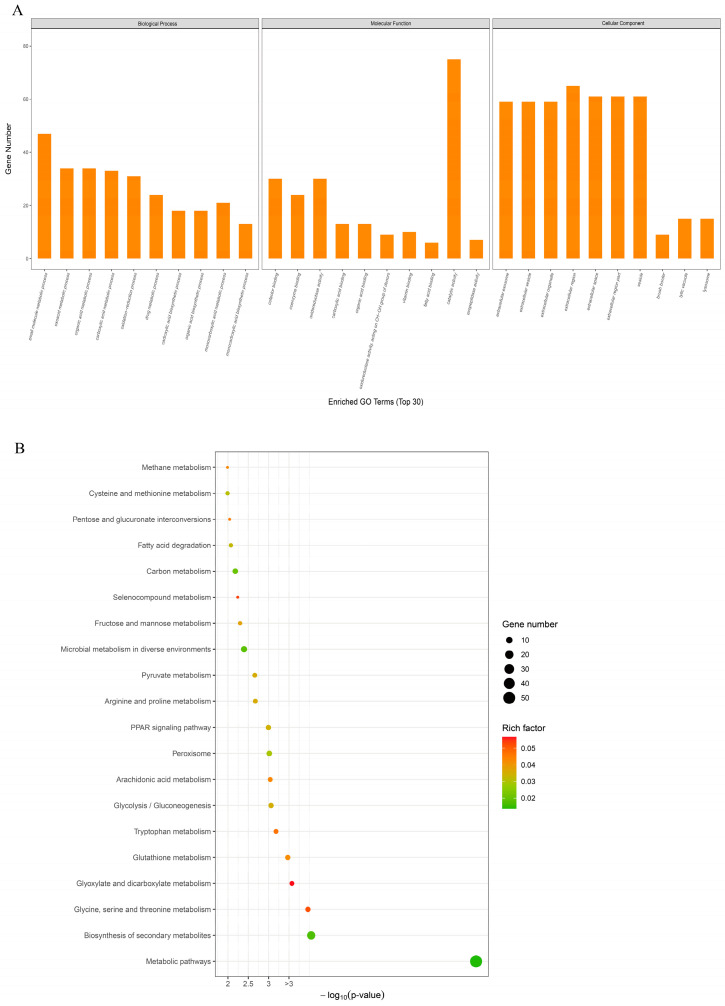
GO annotation of DEGs in *M. rosenbergii* under urea conditions (**A**); KEGG annotation of DEGs in *M. rosenbergii* under urea conditions (**B**).

To better understand biological processes related to DEGs, GO enrichment analysis was performed (Figure 5A). As a result, the DEGs could be categorized into three major types: biological process, molecular function, and cellular component. Small-molecule metabolic processes, oxoacid metabolic processes, and organic acid metabolic processes are the main components of biological processes. The main molecular functions included catalytic activity, oxidoreductase activity, and cofactor binding. The cellular components were associated primarily with extracellular organelles, the extracellular region, and the extracellular space. All DEGs were mapped and annotated in 144 KEGG pathways, of which 30 pathways showed significant enrichment in the urea group in comparison with the control group. Those 20 most significant KEGG pathways are presented in Figure 5B, and are mostly related to amino acid metabolism, like “tryptophan metabolism”, “glycine, serine, and threonine metabolism”, “cysteine and methionine metabolism”, and “arginine and proline metabolism”; the metabolism of other amino acids, such as “glutathione metabolism” and “selenocompound metabolism”; and carbohydrate metabolism, such as “glycolysis/gluconeogenesis”, “glyoxylate and dicarboxylate metabolism”, “pyruvate metabolism”, “fructose and mannose metabolism”, and “pentose and glucuronate interconversion”.

### 3.4. Metabolomics and Transcriptomics Integrated Analysis

**Figure 6:**
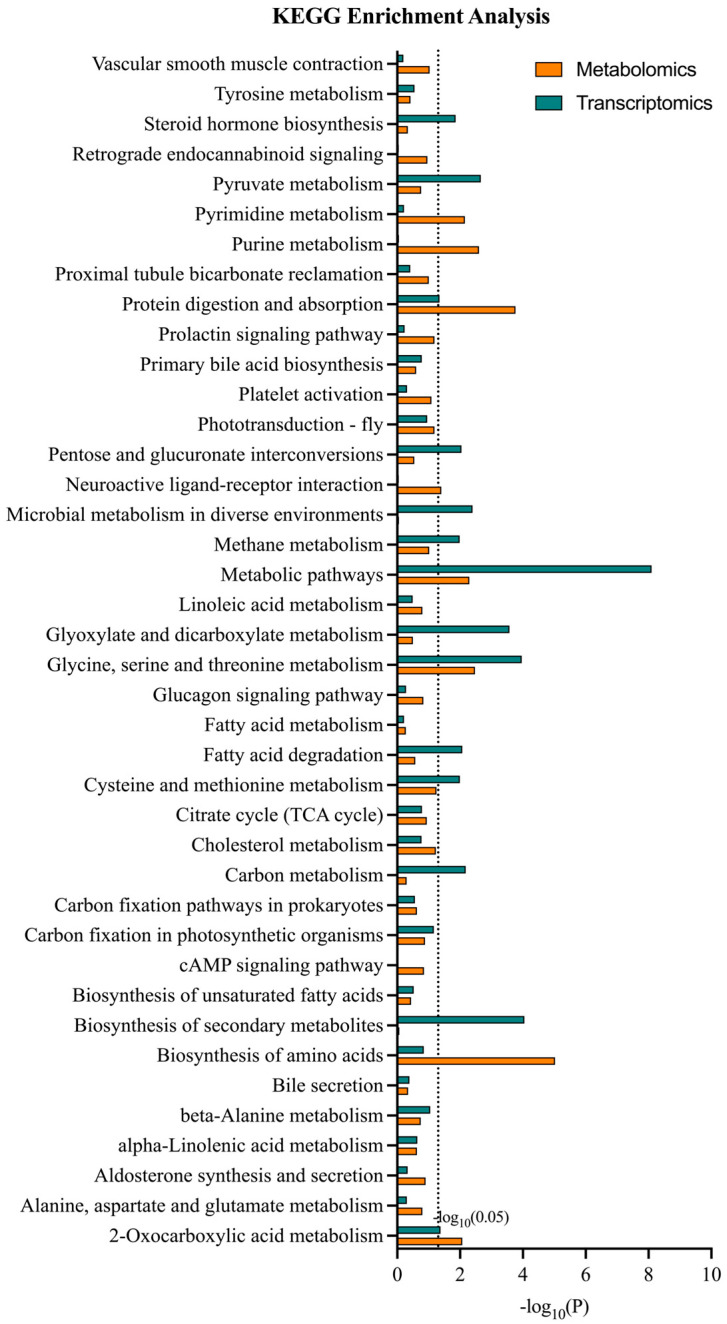
KEGG enrichment analysis of DMs/DEGs in *M. rosenbergii* under urea conditions.

A comparison of pathways associated with genes within transcriptome and metabolites within metabolome revealed 40 co-involved KEGG pathways (Figure 6). Most of the DMs and DEGs were linked to various pathways, like amino acid metabolism, lipid metabolism, and carbohydrate metabolism. Moreover, we compared the transcriptome and metabolic KEGG pathways and investigated the enrichment of several pathways that were significantly different in both categories. The genes and metabolites were markedly related to glycine, serine, and threonine metabolism; 2-oxocarboxylic acid metabolism; protein digestion and absorption; and metabolic pathways. Three DMs and six DEGs, four DMs and two DEGs, and four DMs and four DEGs were associated with glycine, serine, and threonine metabolism, 2-oxocarboxylic acid metabolism, and protein digestion and absorption, respectively.

### 3.5. RT-qPCR Assay of DEGs

**Figure 7:**
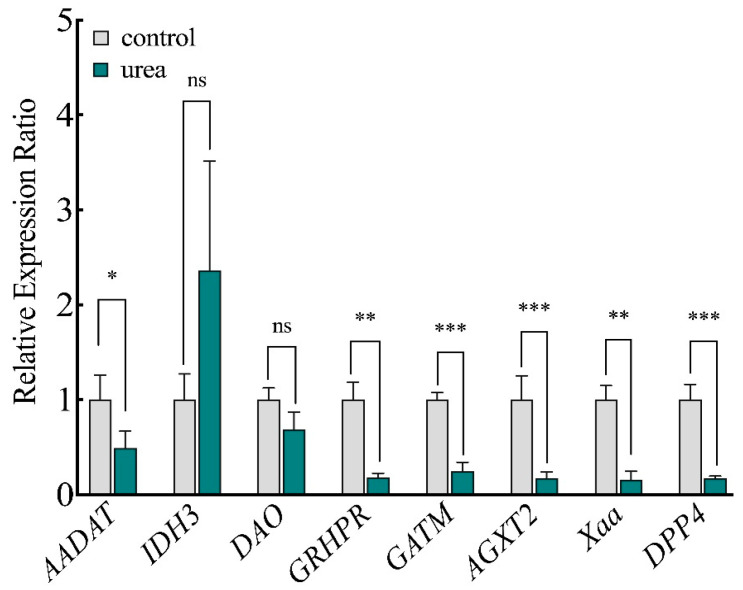
RT-qPCR validation of DEGs. ns: not significant, *: *p* < 0.05, **: *p* < 0.01, ***: *p* < 0.001.

Eight DEGs were subjected to RT-qPCR to confirm that our transcriptomic data were reliable. Seven DEGs were downregulated, and one DEG was upregulated (Figure 7), which matched the transcriptomics results and indicated that the transcriptomics data were reliable.

## 4. Discussion

**Figure 8:**
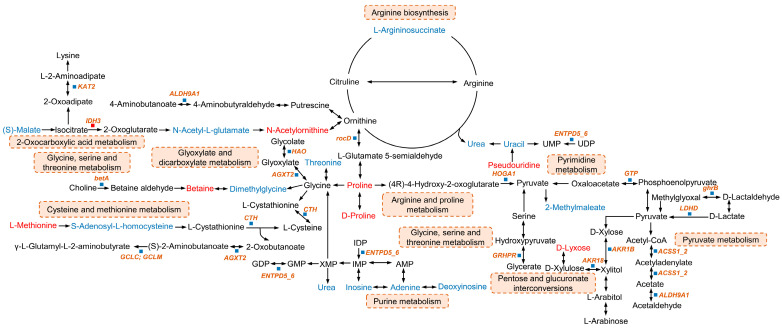
Correlation map of the DMs and DEGs under urea conditions in *M. rosenbergii*. The names of the altered DMs and DEGs are shown in red (upregulated) and blue (downregulated).

The presence of endogenous SEM has caused significant economic losses in aquaculture and aquatic products because it is confused with the illegal addition of NFZ. A study reported that SEM formation in *Litopenaeus vannamei* was related to urea and arginine [4]. The chemical structure of SEM is similar to that of urea [24], and the urea–hydrazine hydrate method for synthesizing SEM is commonly used in industry [25]. Additionally, the use of urea as a source of nitrogen in fertilizers has increased significantly worldwide. Therefore, we hypothesized that endogenous SEM formation in *M. rosenbergii* is related to urea. We first examined the SEM levels in *M. rosenbergii* under urea-induced stress and elucidated the mechanism underlying SEM formation through untargeted metabolomics and transcriptome analysis. The concentration of SEM in *M. rosenbergii* increased with an increase in urea concentration and prolonged exposure to urea, suggesting that urea accelerated endogenous SEM formation within *M. rosenbergii*. This finding was similar to another study in which endogenous SEM formation was found to possibly be associated with urea in crustaceans [4,5]. Urea exerts an important effect on the metabolic processes of organisms, and amino acids arginine and citrulline have a direct relationship to the urea cycle. Yu et al. [4] speculated that endogenous SEM may be produced in the urea cycle, and their speculation was supported by the discovery that arginine, citrulline, and urea can react with hydrogen peroxide to form SEM. Zhang et al. [5] reported that arginine and citrulline can react with urea to form SEM even in the absence of hydrogen peroxide. Additionally, the interaction of arginine and citrulline with amino acids can lead to the formation of polymerized nitrogen, and the amide group may serve as a crucial intermediate in the formation of SEM. These findings indicated that endogenous SEM formation is related to amino acid metabolism, especially the arginine biosynthesis metabolism. L-Argininosuccinate, N-acetyl-L-glutamate, and urea were significantly downregulated, and N-acetylornithine, which participates in arginine biosynthesis and 2-oxocarboxylic acid metabolism, was significantly upregulated (Figure 8). Amino acids are major metabolites involved in biosynthesis and metabolism in many organisms. The metabolites of betaine were significantly upregulated, and those of glycine, serine, and threonine metabolism were significantly downregulated (Figure 8 and Table S3). Moreover, the DEGs related to glycine, serine, and threonine metabolism, including betA (*TR20112_c0_g1*), DAO (*TR3122_c0_g2*), AGXT2 (*TR16673_c0_g1*), GRHPR (*TR95112_c0_g2*), GATM (*TR253_c6_g1*), and CTH (*TR11689_c0_g1*), were downregulated (Figure 8 and Table S5). Protein digestion and absorption are related to amino acid metabolism. Threonine is downregulated, whereas proline and L-methionine are upregulated in protein digestion and absorption, which also influence cysteine and methionine metabolism, along with glycine, serine, and threonine metabolism. The DEGs related to protein digestion and absorption, including Xaa (*TR61262_c0_g1*), MME (*TR87543_c0_g1*), SLC3A1 (*TR11680_c0_g2*), and DPP4 (*TR8552_c0_g1*), were downregulated. Glycine, serine, and threonine metabolism and protein digestion and absorption were disrupted in the urea group, which indicated that amino acid metabolism was impaired. Some studies found the dysregulation of glycine, serine, and threonine metabolism and protein digestion within nitrogenous compound-stressed Nile tilapia and *L. vannamei* [26,27]. Most of the DEGs and DMs were found to participate in arginine biosynthesis through ornithine, thus participating in the urea cycle. Therefore, we hypothesized that disrupting these metabolic pathways can affect arginine-related metabolisms, leading to endogenous SEM formation.

Additionally, as revealed by untargeted metabolomics analysis, nucleotide metabolism was also significantly affected in the urea group, and most of the DMs were enriched in nucleotide metabolism, including purine and pyrimidine metabolism. Nucleotide metabolism is closely related to amino acid metabolism. In this study, uracil was significantly downregulated through the urea involved in arginine biosynthesis metabolism. Four DMs (urea, inosine, adenine, and deoxyinosine) and one DEG (ENTPD5_6 (*TR5309_c1_g2*)) were downregulated in purine metabolism, which indicated that *M. rosenbergii* resists urea conditions by regulating energy metabolism. A similar phenomenon was observed in sea bream, where environmental stress disrupted purine metabolism [28]. Besides amino acid and nucleotide metabolism, most DEGs and KEGG pathways identified from transcriptome analysis were also associated with carbohydrate metabolism. These findings indicated that urea disrupts carbohydrate metabolism in *M. rosenbergii*. Other studies have found that carbohydrate metabolism serves as the primary energy source that helps aquatic animals resist stress [29,30]. These results indicated that urea induces the formation of endogenous SEM in *M. rosenbergii*, primarily by impairing amino acid-, nucleotide-, and carbohydrate-related metabolic pathways, especially the pathway related to arginine.

## 5. Conclusions

The present work first reported that significant endogenous SEM levels in *M. rosenbergii* were formed in the presence of urea. A dose-dependent relation of SEM concentration with both urea concentration and stress duration could be detected. The mechanism related to endogenous production of SEM in *M. rosenbergii* under urea conditions was explored by omics analysis. The results of integrated analysis of metabolome and transcriptome data suggested significant disturbances in glycine, serine, and threonine metabolism; 2-oxocarboxylic acid metabolism; and protein digestion and absorption. These findings provide new insights into endogenous SEM production in *M. rosenbergii*. Additionally, future studies should focus on endogenous SEM in crustaceans and investigate in depth the mechanism underlying endogenous SEM formation in crustaceans in a urea environment. The present work offers novel information for assessing the safety and quality of aquatic products.

## Data Availability

The original contributions presented in this study are included in the article/Supplementary Materials. Further inquiries can be directed to the corresponding author.

## Supplementary Materials

The following supporting information can be downloaded at: https://www.mdpi.com/article/10.3390/foods13233817/s1. Table S1: Gradient elution program of liquid chromatography; Table S2: Semicarbazide and internal target mass spectrometry conditions; Table S3: The DMs in control versus urea groups; Table S4: Overall evaluation of sequencing date in transcriptome; Table S5: The DEGs in control versus urea groups; Table S6: Primers of RT-qPCR validation [31].
